# 963 Updated Epidemiology of Pediatric Burn Injuries Under 10% Total Body Surface Area: A Five-Year Review

**DOI:** 10.1093/jbcr/iraf019.494

**Published:** 2025-04-01

**Authors:** Jessica Willoughby, Delaney Moslander, Kathy Prelack

**Affiliations:** Shriners Children’s - Boston; Shriners Children’s - Boston; Shriners Children’s - Boston

## Abstract

**Introduction:**

The trends of pediatric burn injuries have remained consistent over the years, however given non-fatal burns are often treated by non-burn-specific providers an updated review of this population was conducted. This five-year review provides public health and medical providers an updated look at the current demographics, etiology, and acute wound management to promote public education and prevention of burn injuries. This study examined the demographics, burn characteristics, and incidence of hypertrophic scarring among this population.

**Methods:**

A retrospective review was conducted on 2,684 pediatric patients treated at an outpatient clinic of an ABA-verified pediatric burn center who experienced a burn injury under 10% TBSA between 1/1/2015 and 1/1/2020. Descriptive statistics were used to analyze the frequency of the variables which included age, race and ethnicity, Fitzpatrick skin type, mechanism of injury, location of injury, size and depth of injury, acute wound management, days to wound closure, involvement of therapy services, and development of hypertrophic scarring.

**Results:**

The majority of burns were scalds (50.63%) and partial thickness (97.40%). The palmar hand (22.18%) was the most common location. Most of the injuries healed within 8-14 days (40.90%) within this study. The average patient age was 4.78 years with an average TBSA of 1.33%. Fitzpatrick skin type 2 was most observed (40.87%) and 19.20% of the population identified as Hispanic. Families reported that 38.07% of burn injuries were cooled with water and 35.99% were not cooled at all. The rehabilitation department assessed 21.01% of patients and 10.17% developed hypertrophic scarring. Nearly 25% of patients stopped attending appointments while a wound was still open.

**Conclusions:**

The number of patients in this study who developed hypertrophic scarring was much lower than previous studies which report rates between 32 and 71%. Scalds continue to be the most common mechanism of burn injury in the pediatric population, with almost all injuries sustained being preventable ones.

**Applicability of Research to Practice:**

Although burn injuries under 10% TBSA are small, they still have a large impact on a child’s quality of life and warrant further research. Nearly all burn injuries are preventable and despite best efforts in burn prevention and acute wound management there continues to be a lack of public knowledge. Additional education efforts in these areas should be made as these injuries are often initially treated by non-burn care-specific providers. Small burn injuries may not be referred to a verified burn center at all yet can greatly impact function. This further indicates the need for additional education and efforts in managing burns of all sizes.

**Funding for the Study:**

N/A

